# Medicinal Plant Root Exudate Metabolites Shape the Rhizosphere Microbiota

**DOI:** 10.3390/ijms25147786

**Published:** 2024-07-16

**Authors:** Peng Qu, Butian Wang, Meijun Qi, Rong Lin, Hongmei Chen, Chun Xie, Zhenwei Zhang, Junchao Qiu, Huabo Du, Yu Ge

**Affiliations:** College of Tropical Crops, Yunnan Agricultural University, Pu’er 665099, China; qupeng1988@163.com (P.Q.); wangbutian@stu.ynau.edu.cn (B.W.); qimeijun2003@163.com (M.Q.); linrong0529@163.com (R.L.); 18187965067@163.com (H.C.); xiechun2008077@163.com (C.X.); zhangzhenwei202309@163.com (Z.Z.); qiujunchao23@163.com (J.Q.)

**Keywords:** root exudates, rhizosphere microbes, plant species

## Abstract

The interactions between plants and rhizosphere microbes mediated by plant root exudates are increasingly being investigated. The root-derived metabolites of medicinal plants are relatively diverse and have unique characteristics. However, whether medicinal plants influence their rhizosphere microbial community remains unknown. How medicinal plant species drive rhizosphere microbial community changes should be clarified. In this study involving high-throughput sequencing of rhizosphere microbes and an analysis of root exudates using a gas chromatograph coupled with a time-of-flight mass spectrometer, we revealed that the root exudate metabolites and microorganisms differed among the rhizosphere soils of five medicinal plants. Moreover, the results of a correlation analysis indicated that bacterial and fungal profiles in the rhizosphere soils of the five medicinal plants were extremely significantly or significantly affected by 10 root-associated metabolites. Furthermore, among the 10 root exudate metabolites, two (carvone and zymosterol) had opposite effects on rhizosphere bacteria and fungi. Our study findings suggest that plant-derived exudates modulate changes to rhizosphere microbial communities.

## 1. Introduction

Plants provide multiple nutrient-rich microhabitats for microorganisms at the soil–root interface, which is divided into the rhizosphere, rhizoplane, and endosphere from outside to inside the roots [1]. The rhizosphere is the soil microdomain around the roots that is most affected by root exudates [2]. The rhizosphere contains a wide variety of eukaryotic and prokaryotic microbial cells [3,4]. These microbial interactions influence biogeochemical cycles, plant growth, and plant tolerance to biotic and abiotic stresses. Because the total genome of the plant rhizosphere microbial community is much larger than the total genome of the host plant, it is also called the second genome of the plant [5]. Rhizosphere microorganisms mainly consist of soil microorganisms, with specific taxa that enter the root system [6]. The rhizosphere microbial groups mainly include bacteria, fungi, viruses, protists, and microalgae, among which bacteria and fungi have been more extensively studied [7]. The main rhizosphere bacteria are from the phyla Proteobacteria, Acidobacteria, and Actinobacteria, whereas the main rhizosphere fungi are from the phyla Basidiomycota and Ascomycota [8].

The rhizosphere microbial community composition is greatly influenced by the host plant. For example, the rhizosphere microbial communities of alfalfa and soybean differ regarding composition, with the same soil physicochemical indices differentially influencing alfalfa and soybean communities [9]. Earlier research detected significant differences in the multigenerational rhizosphere microorganisms in three model plants (*Arabidopsis thaliana*, alfalfa, and yellow flower orchid) and three crops (Chinese cabbage, pea, and wheat) [10]. Tkacz et al. [11] also compared the microbiota community structures of four different plant species (*A. thaliana*, *Medicago truncatula*, *Pisum sativum*, and *Triticum aestivum*) grown under the same soil conditions, which revealed considerable differences in the prokaryotic and eukaryotic communities of the plant-associated fractions. However, the effect of host plants on rhizosphere microbial communities is largely related to root metabolites [12].

Approximately 40% of the carbon absorbed by plants during photosynthesis is transmitted to the soil in the form of compounds secreted by the roots [13], thereby leading to environmental changes. These root exudates contain primary metabolites, such as amino acids, carbohydrates, organic acids, and plant hormones, as well as secondary metabolites, including flavonoids, terpenes, auxin, and alkaloids [14]. Plant root exudates have substantial effects on the rhizosphere soil and microbial community [15]. Because root exudates are released into the rhizosphere soil, the microbial community is much larger in the rhizosphere soil than in the surrounding soil [16]. By studying the rhizosphere microbial community of the *f6h1 A. thaliana* mutant lacking a coumarin synthesis pathway, Voges et al. [17] determined that coumarin can inhibit the growth of *Pseudomonas* species via a mechanism mediated by redox reactions. Huang et al. [18] analyzed the triterpenoid synthesis network in *A. thaliana*, which can synthesize more than 50 unknown root exudates, while also examining the effects of purified root exudates on 10 microorganisms isolated from *A. thaliana* in in vitro experiments. These compounds can selectively regulate bacterial growth, with some bacteria selectively metabolizing certain triterpenoids (e.g., thalianyl fatty acid esters). Neal et al. [19] determined that the maize root system secretes benzoxazines, which attract *Pseudomonas putida* to the rhizosphere. Similarly, in tomato, banana, watermelon, and *A. thaliana*, other types of organic acids can recruit plant growth-promoting rhizogenic bacteria [20]. Aromatic organic acids secreted by plants (niacin, shikimic acid, cinnamic acid, salicylic acid, and indole-3-acetic acid (IAA)) are more attractive to rhizosphere bacteria [21].

In medicinal plants, long-term selective cultivation has increased the secondary metabolite contents in the roots compared to other plants, which increases the release of metabolites into the rhizosphere from the roots [22]. China’s Yunnan Province has the largest plantation area of rubber forest, and *Alpinia katsumadai*, *Amomum villosum*, *Alpinia officinarum*, *Alpinia oxyphylla*, and *Baphicacanthus cusia* are the five most common medicinal plants planted under the rubber forest in Yunnan Province in China. Hence, in this study, five main medicinal plants (*Alpinia katsumadai*, *Amomum villosum*, *Alpinia officinarum*, *Alpinia oxyphylla*, and *Baphicacanthus cusia*) in a rubber forest were used to analyze the effects of root exudate metabolites on rhizosphere microorganisms among plant species and root parts. The objectives of this study were as follows: (i) to analyze and compare the root exudate metabolites and rhizosphere microbiota community structures of the five different medicinal plants (*A. katsumadai*, *A. villosum*, *A. officinarum*, *A. oxyphylla*, and *B. cusia*) in a rubber forest; (ii) to assess the correlation between root exudate metabolites and rhizosphere microbiota. Overall, we determined that plant species is the main factor driving rhizosphere microbial community changes mediated by root exudate metabolites.

## 2. Results

### 2.1. Bacterial and Fungal Communities in the Rhizosphere Soils of A. katsumadai, A. villosum, A. officinarum, A. oxyphylla, B. cusia, and the Pure Rubber Forest

**Figure 1:**
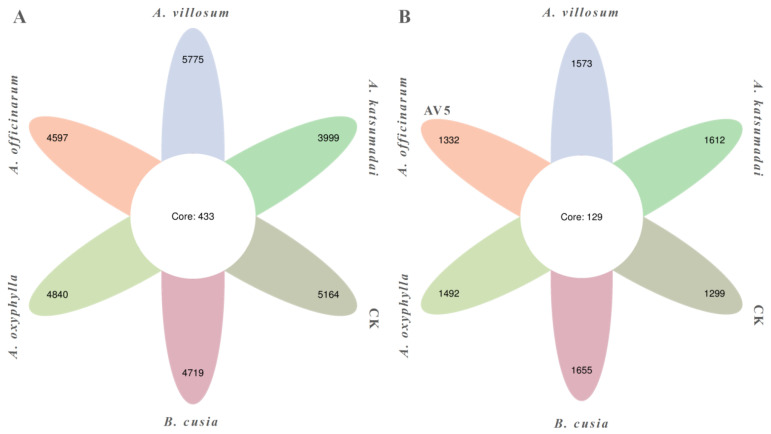
Venn diagram of the bacterial and fungal OTUs for the rhizosphere soils of *A. katsumadai*, *A. villosum*, *A. officinarum*, *A. oxyphylla, B. cusia*, and the pure rubber forest. (**A**) Venn diagram of the bacterial OTUs. (**B**) Venn diagram of the fungal OTUs. CK represents the soil sample from the pure rubber forest.

A total of 29,094 bacterial operational taxonomic units (OTUs) (97% similarity) were obtained, with *A. katsumadai, A. villosum, A. officinarum, A. oxyphylla, B. cusia,* and CK (the soil sample from the pure rubber forest) rhizosphere soil samples containing 3999, 5775, 4597, 4840, 4719, and 5164 bacterial OTUs, respectively. There were 433 shared OTUs in the six sample groups, accounting for 1.49% of the bacterial OTUs in all sample groups, reflecting the considerable differences in the bacteria in the six sample groups (Figure 1A). The rank order of the ratio of the number of shared OTUs to the number of all OTUs in pairwise comparisons was as follows: *A. officinarum* vs. *B. cusia* (24.93%) > *A. katsumadai* vs. *B. cusia* (23.69%) > *A. officinarum* vs. *A. oxyphylla* (23.28%) > *A. oxyphylla* vs. *B. cusia* (23.10%) > *A. katsumadai* vs. *A. oxyphylla* (19.82%) > *A. oxyphylla* vs. CK (19.69%) > *A. katsumadai* vs. *A. officinarum* (19.41%) > *B. cusia* vs. CK (18.42%) > *A. villosum* vs. *A. oxyphylla* (17.18%) > *A. officinarum* vs. CK (16.13%) > *A. katsumadai* vs. CK (14.93%) > *A. villosum* vs. CK (14.62%) > *A. villosum* vs. *A. officinarum* (12.40%) > *A. villosum* vs. *B. cusia* (12.38%) > *A. katsumadai* vs. *A. villosum* (9.61%) (Table S1). Statistical tables of valid data of bacterial OTUs in the rhizosphere soils of *A. katsumadai*, *A. villosum*, *A. officinarum*, *A. oxyphylla*, *B. cusia*, and CK are listed in Table S2.

A total of 8963 fungal OTUs (97% similarity) were obtained, with the *A. katsumadai*, *A. villosum*, *A. officinarum*, *A. oxyphylla*, *B. cusia*, and CK rhizosphere soil sample groups containing 1612, 1573, 1332, 1492, 1655, and 1299 fungal OTUs, respectively. There were 129 shared OTUs in the six sample groups, accounting for 1.44% of the fungal OTUs in all sample groups, indicative of the substantial differences in the fungi in the six sample groups (Figure 1B). The rank order of the ratio of the number of shared OTUs to the number of all OTUs in pairwise comparisons was as follows: *A. officinarum* vs. *A. oxyphylla* (19.41%) > *A. officinarum* vs. *B. cusia* (18.81%) > *A. oxyphylla* vs. CK (18.46%) > *A. oxyphylla* vs. *B. cusia* (18.09%) > *A. katsumadai* vs. *B. cusia* (17.43%) > *A. officinarum* vs. CK (16.67%) > *A. katsumadai* vs. *A. officinarum* (16.59%) > *A. katsumadai* vs. *A. oxyphylla* (16.17%) > *B. cusia* vs. CK (15.21%) > *A. villosum* vs. CK (14.97%) > *A. villosum* vs. *A. oxyphylla* (17.79%) > *A. villosum* vs. *A. officinarum* (13.92%) > *A. katsumadai* vs. CK (13.36%) > *A. villosum* vs. *B. cusia* (13.22%) > *A. katsumadai* vs. *A. villosum* (10.82%) (Table S3). Statistical tables of valid data of fungal OTUs in the rhizosphere soils of *A. katsumadai*, *A. villosum*, *A. officinarum*, *A. oxyphylla*, *B. cusia*, and CK are listed in Table S4.

**Figure 2:**
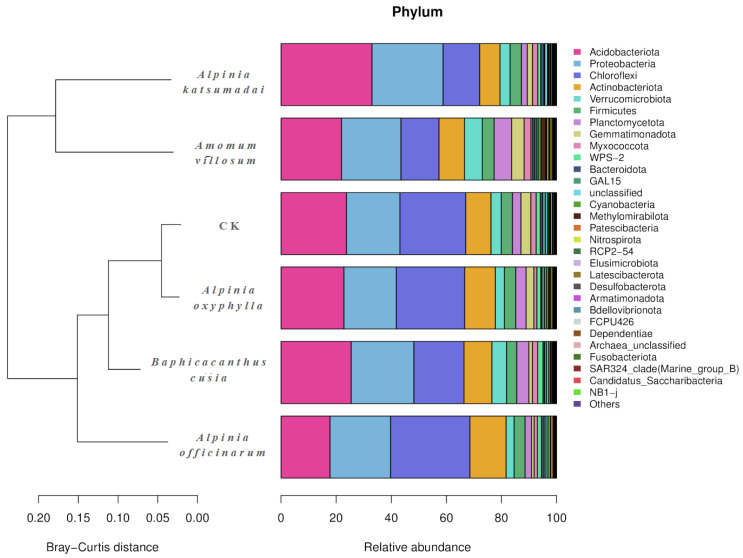
Bacterial community composition and structure in the rhizosphere soils of *A. katsumadai*, *A. villosum*, *A. officinarum*, *A. oxyphylla, B. cusia*, and the pure rubber forest. CK represents the soil sample from the pure rubber forest.

The 16S rRNA V3–V4 region of the bacterial communities in the six rhizosphere soil sample groups was sequenced and classified (including no-rank and unclassified). A total of 3 kingdoms, 31 phyla, 146 classes, 321 orders, 535 families, 1075 genera, and 1377 species were detected in 18 rhizosphere soil samples. The three kingdoms were the bacterial kingdom (99.21%), the archaea kingdom (0.07%), and the unclassified kingdom (0.72%). At the bacterial phylum level (Figure 2), the dominant phyla (mean relative abundance ≥10%) in all samples were Acidobacteria (24.11%), Proteobacteria (21.77%), Chloroflexi (20.46%), and Actinobacteriota (10.03%). The rhizosphere soil samples of *A. katsumadai* had the highest relative abundances of Acidobacteriota and Proteobacteria, but the lowest relative abundances of Chloroflexi and Actinobacteriota. In contrast, the rhizosphere soil samples of *A. officinarum* had the highest relative abundances of Chloroflexi and Actinobacteriota, but the lowest relative abundance of Acidobacteriota. The rhizosphere soil samples of *A. oxyphylla* had the lowest relative abundance of Proteobacteria. The relative abundances (%) of the bacteria at the phylum level in the rhizosphere soils of *A. katsumadai*, *A. villosum*, *A. officinarum*, *A. oxyphylla*, *B. cusia*, and CK are listed in Table S5.

**Figure 3:**
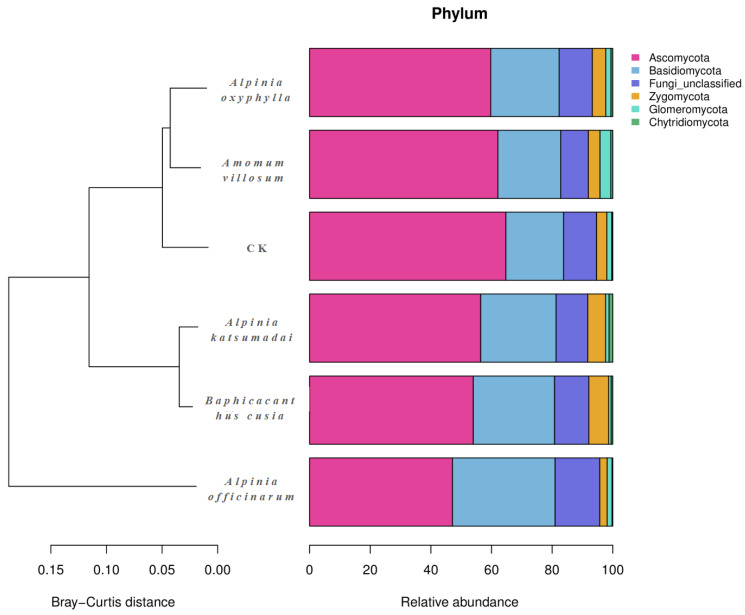
Fungal community composition and structure in the rhizosphere soils of *A. katsumadai*, *A. villosum*, *A. officinarum*, *A. oxyphylla, B. cusia*, and the pure rubber forest. CK represents the soil sample from the pure rubber forest.

The OTUs present in the ITS1-1 F regions of the fungal ITS sequences in 18 soil samples were sequenced and classified (including no-rank and unclassified), resulting in the detection of 1 kingdom, 6 phyla, 28 classes, 75 orders, 170 families, 355 genera, and 629 species. The soil fungal community mainly included three dominant phyla (mean relative abundance ≥10%; Figure 3), which were Ascomycota (57.35%), Basidiomycota (24.65%), and Fungi_unclassified (11.23%). The rhizosphere soil samples of *A. officinarum* had the highest relative abundances of Basidiomycota and Fungi_unclassified, but the lowest relative abundance of Ascomycota. In contrast, the rhizosphere soil samples of CK had the highest relative abundance of Ascomycota, but the lowest relative abundance of Basidiomycota. The rhizosphere soil samples of *A. villosum* had the lowest relative abundance of Fungi_unclassified. The relative abundances (%) of the fungi at the phylum level in the rhizosphere soils of *A. katsumadai*, *A. villosum*, *A. officinarum*, *A. oxyphylla*, *B. cusia*, and CK are listed in Table S6.

A *T* test was used to screen for the dominant bacterial phyla (mean relative abundance ≥10%), with significant differences in relative abundance among the six rhizosphere soil sample groups determined on the basis of pairwise comparisons (Table 1). The relative abundance of Acidobacteria differed significantly among the 12 comparisons. For Chloroflexi and Actinobacteriota, significant differences in relative abundance were detected in 11 comparisons. Differences in the relative abundance of Proteobacteria were revealed by nine comparisons.

A *T* test was used to identify the dominant fungal phyla (mean relative abundance ≥10%), with significant differences in relative abundance among the six rhizosphere soil sample groups determined on the basis of pairwise comparisons. The relative abundance of Ascomycota varied significantly in five comparisons (Table 2). Compared with the CK rhizosphere soil, the rhizosphere soil samples of *A. katsumadai* and *B. cusia* had a significantly lower relative abundance of Ascomycota. The relative abundance of Ascomycota was significantly higher in the CK, *A. oxyphylla*, and *A. villosum* rhizosphere soils than in the rhizosphere soil sample of *A. officinarum*. The relative abundance of Basidiomycota was significantly higher in the *A. officinarum* rhizosphere soil than in the CK rhizosphere soil (Table 2).

### 2.2. Microbial Diversity in the Rhizosphere Soils of A. katsumadai, A. villosum, A. officinarum, A. oxyphylla, B. cusia, and the Pure Rubber Forest

#### 2.2.1. Alpha Diversity in the Rhizosphere Soils of *A. katsumadai*, *A. villosum*, *A. officinarum*, *A. oxyphylla*, *B. cusia*, and the Pure Rubber Forest

Alpha diversity indices (i.e., Observed_OTUs, Shannon, Simpson, Chao1, Goods_coverage, and Pielou_e) were used to reveal the bacterial community diversity in the six rhizosphere soils (Table 3). The richness (Observed_OTUs and Chao1) of rhizosphere bacterial communities did not differ significantly among the six rhizosphere soils (*p* < 0.05). There were significant differences in the Shannon index of the bacterial communities of the six rhizosphere soils (*p* < 0.05), but there were almost no differences in the Simpson index of the bacterial communities in the six rhizosphere soils. The bacterial coverage rate (Goods_coverage) indicated that the sequencing results of all samples perfectly represented the real situations of the samples, and the probability that a new species was not detected in the samples was statistically zero. The evenness index (Pielou_e) of the rhizosphere bacterial communities in all samples was high and did not differ substantially among samples.

Alpha diversity indices (i.e., Observed_OTUs, Shannon, Simpson, Chao1, Goods_coverage, and Pielou_e) were used to reveal the fungal community diversity in the six rhizosphere soils (Table 4). The richness (Observed_OTUs and Chao1) of the rhizosphere fungal communities did not differ significantly among the six rhizosphere soils (*p* < 0.05). Similarly, there were no significant differences in the diversity (Shannon and Simpson) of the rhizosphere fungal communities among the six rhizosphere soils (*p* < 0.05). The fungal coverage rate (Goods_coverage) indicated that the sequencing results of all samples perfectly represented the real situations of the samples, and the probability that a new species was not detected in the samples was statistically zero. The evenness index (Pielou_e) of the rhizosphere fungal communities in all samples was high, with no major differences among samples.

#### 2.2.2. Beta Diversity Indices of the Bacterial and Fungal Communities in the Rhizosphere Soils of *A. katsumadai*, *A. villosum*, *A. officinarum*, *A. oxyphylla*, *B. cusia*, and the Pure Rubber Forest

**Figure 4:**
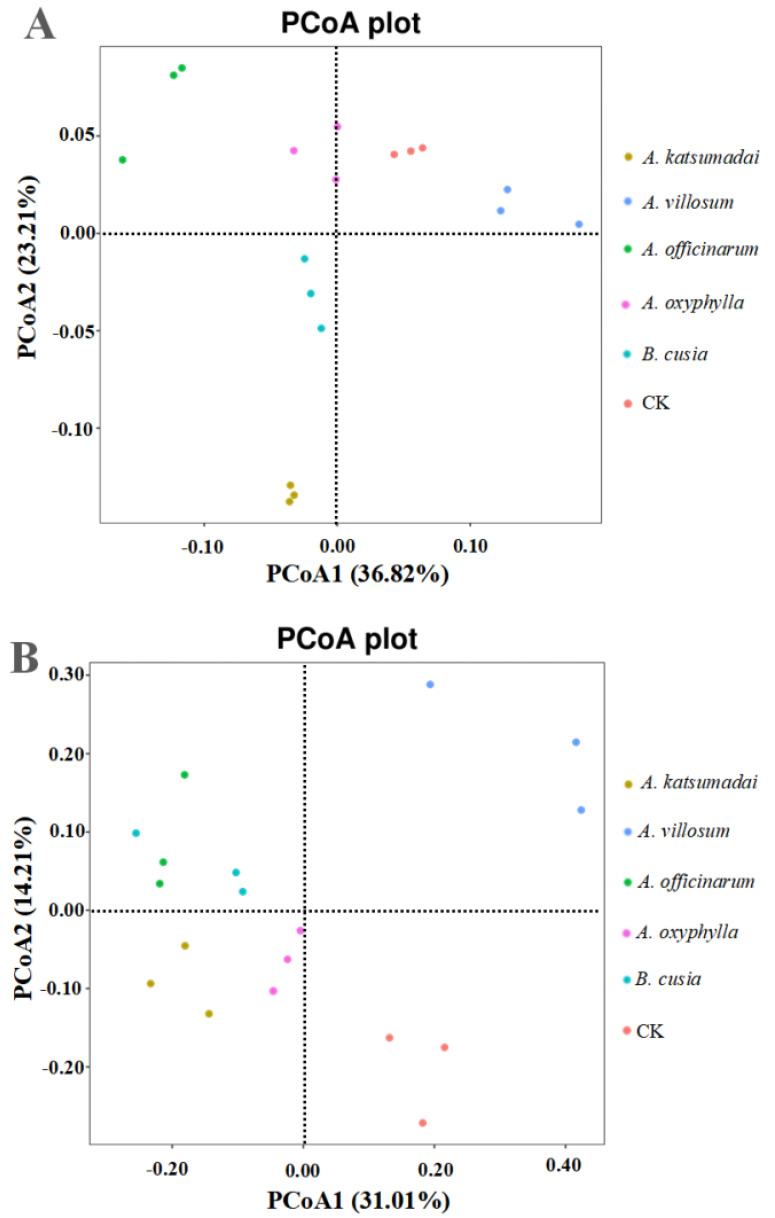
Principle coordinate analysis (PCoA) of the bacterial and fungal communities in the rhizosphere soils of *A. katsumadai*, *A. villosum*, *A. officinarum*, *A. oxyphylla, B. cusia*, and the pure rubber forest. (**A**) PCoA of the bacterial community. (**B**) PCoA of the fungal community. CK represents the soil sample from the pure rubber forest.

On the basis of the first principal component (36.82% contribution), the bacterial communities of the *A. villosum* and CK rhizosphere soils were clustered in the same axis, separate from the bacterial communities of the other four rhizosphere soils (Figure 4A). The bacterial communities in the *A. officinarum* and *A. oxyphylla* rhizosphere soils were distinguished from the bacterial communities in the *A. katsumadai* and *B. cusia* rhizosphere soils by the second principal component (23.21% contribution). Figure 4B presents the distribution of the rhizosphere fungal communities among the six rhizosphere soils for the two principal axes of variation, as determined by principal coordinate analysis (PCoA). On the basis of the first coordinate, which accounted for 31.01% of the total variation, the fungal communities of the *A. villosum* and CK rhizosphere soils were grouped separately from the fungal communities of the other four rhizosphere soils. On the basis of the second coordinate, which accounted for 14.21% of the total variation, the bacterial communities of the *A. officinarum* and *B. cusia* rhizosphere soils were generally grouped separately from the bacterial communities of the *A. katsumadai* and *B. cusia* rhizosphere soils.

### 2.3. Root Exudate Metabolites in the Rhizosphere Soils of A. katsumadai, A. villosum, A. officinarum, A. oxyphylla, B. cusia, and the Pure Rubber Forest

A GC-TOF-MS system was used to analyze the root exudate metabolites in the rhizosphere soils of *A. katsumadai*, *A. villosum*, *A. officinarum*, *A. oxyphylla, B. cusia*, and CK. A comparison of the mass spectra of the analytes with those of commercial reference standard compounds resulted in the identification of 223 root exudate metabolites in the rhizosphere soils of *A. katsumadai*, *A. villosum*, *A. officinarum*, *A. oxyphylla, B. cusia*, and CK (Table S7). The rank order of the proportion of root exudate metabolites in 11 superclasses among the six rhizosphere soils was as follows: organic acids and derivatives (25.11%) > lipids and lipid-like molecules (21.08%) > organic oxygen compounds (20.18%) > others (14.35%) > organoheterocyclic compounds (7.17%) > benzenoids (4.04%) > nucleosides, nucleotides, and analogs (2.69%) = phenylpropanoids and polyketides (2.69%) > organic nitrogen compounds (1.79%) > hydrocarbons (0.45%) = lignans, heolignans, and related compounds (0.45%). The data were subjected to a principal component analysis (PCA), which clearly separated the six rhizosphere soils in the PC1 (18.70% contribution) × PC2 (13.00% contribution) score plot (Figure S1). On the basis of the first principal component, the root exudate metabolites of the *A. katsumadai*, *A. officinarum*, *A. oxyphylla*, and CK rhizosphere soils were grouped separately from the root exudate metabolites of the *A. villosum* and *B. cusia* rhizosphere soils. The root exudate metabolites in the rhizosphere soils of *A. katsumadai*, *A. officinarum*, and *A. oxyphylla* were clustered together in the same quadrant. Moreover, they were distinguished from the root exudate metabolites of the CK rhizosphere soil on the basis of the second principal component. Similarly, the root exudate metabolites of the *A. villosum* and *B. cusia* rhizosphere soils were scattered in different quadrants according to the second principal component.

During pairwise comparisons, significant differences between root exudate metabolites were determined using the following criteria: variable importance in projection > 1 and *p* < 0.05 (Table S8). There were significant differences in the relative contents of 17–100 root exudate metabolites among the 15 groups (Table 5). Of these pairwise comparisons, *B. cusia* vs. *A. officinarum* had the fewest root exudate metabolites with significant differences (17), whereas CK vs. *A. oxyphylla* had the most root exudate metabolites with significant differences (100). The main differentially abundant root exudate metabolites were lipids and lipid-like molecules and organic acids and derivatives (Table S8). One to five root exudate metabolites had relative contents that differed between rhizosphere soils by a factor of 1000 (Table 5).

### 2.4. Metabolic Pathway Analysis of Differentially Abundant Root Exudate Metabolites in Pairwise Comparisons of the Rhizosphere Soils of A. katsumadai, A. villosum, A. officinarum, A. oxyphylla, B. cusia, and the Pure Rubber Forest

In the present study, the differential abundance (DA) score revealed differences in the metabolic pathways associated with the root exudate metabolites in pairwise comparisons of the rhizosphere soils of *A. katsumadai*, *A. villosum*, *A. officinarum*, *A. oxyphylla*, *B. cusia*, and the pure rubber forest (Figure S2). The DA score, which is the ratio between the number of up-regulated and down-regulated differentially abundant metabolites associated with a pathway and the number of all metabolites associated with the pathway, can reflect the overall changes in the differentially abundant metabolites associated with a pathway.

Extremely significant differences and the most root exudate metabolites associated with metabolic pathways were detected in the *A. villosum* vs. *A. katsumadai*, *A. oxyphylla* vs. *A. katsumadai*, *A. officinarum* vs. *A. katsumadai*, CK vs. *A. katsumadai*, *A. officinarum* vs. *A. villosum*, *A. oxyphylla* vs. *A. villosum*, CK vs. *A. villosum*, *A. oxyphylla* vs. *A. officinarum*, CK vs. *A. officinarum*, CK vs. *A. oxyphylla*, and CK vs. *B. cusia* comparisons. Extremely significant differences and the most ABC transporter-related root exudate metabolites were revealed by the *B. cusia* vs. *A. katsumadai* and *B. cusia* vs. *A. oxyphylla* comparisons. Extremely significant differences and the most root exudate metabolites related to glycine, serine, and threonine metabolism were detected in the *B. cusia* vs. *A. villosum* comparison. Additionally, extremely significant differences and the most root exudate metabolites associated with steroid hormone biosynthesis and ABC transporters were revealed by the *B. cusia* vs. *A. officinarum* comparison.

### 2.5. Correlation between Root Exudate Metabolites and Rhizosphere Microbiota

**Figure 5:**
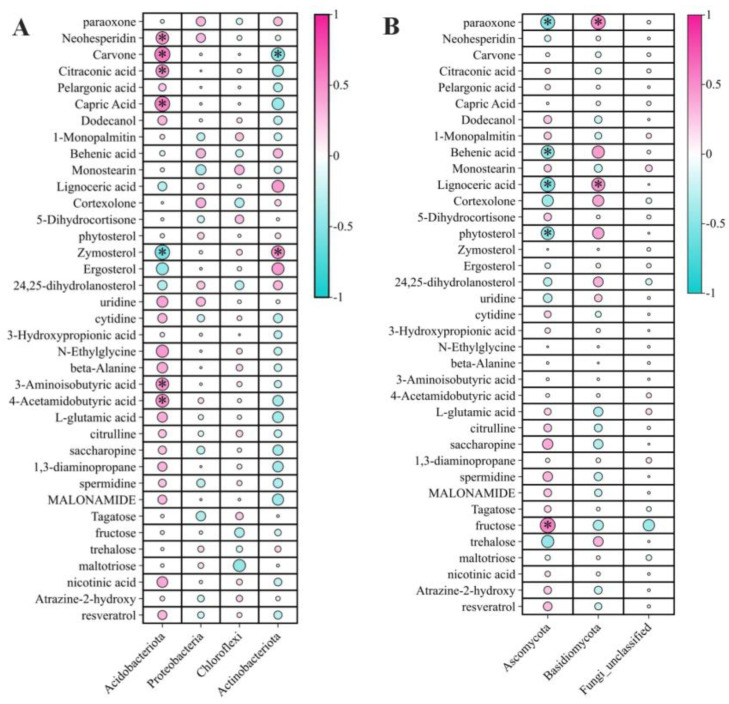
Correlations between root exudate metabolites and the microbiota of the rhizosphere soils of *A. katsumadai*, *A. villosum*, *A. officinarum*, *A. oxyphylla*, *B. cusia*, and the pure rubber forest. (**A**) Correlations between the 37 differentially abundant root exudate metabolites and four dominant rhizosphere bacterial phyla. (**B**) Correlations between the 37 differentially abundant root exudate metabolites and three dominant rhizosphere fungal phyla. * significant difference (*p* < 0.05); ** extremely significant difference (*p* < 0.01).

During a multi-group comparison, significant differences in the root exudate metabolites of the six rhizosphere soils were determined using the following criteria: ANOVA *p* < 0.01 and *q* < 0.01. The 37 root exudate metabolites that differed significantly among the six rhizosphere soils were from the following nine superclasses: benzenoids (1), lignans, neolignans, and related compounds (1), lipids and lipid-like molecules (14), nucleosides, nucleotides, and analogs (2), organic acids and derivatives (8), organic nitrogen compounds (2), organic oxygen compounds (5), organoheterocyclic compounds (2), and phenylpropanoids and polyketides (1) (Table S9). Correlations between the 37 differentially abundant root exudate metabolites and the dominant rhizosphere bacterial and fungal phyla are presented in Figure 5. Among the four dominant rhizosphere bacterial phyla, Acidobacteria was significantly positively correlated with neohesperidin (r = 0.47), carvone (r = 0.58), citraconic acid (r = 0.48), capric acid (r = 0.48), 3-aminoisobutyric acid (r = 0.47), and 4-acetamidobutyric acid (r = 0.48), but significantly negatively correlated with zymosterol (r = −0.55). Additionally, Actinobacteriota was significantly positively correlated with zymosterol (r = 0.49), but significantly negatively correlated with carvone (r = −0.48). Among the three dominant rhizosphere fungal phyla, Ascomycota was significantly negatively correlated with paraoxone (r = −0.52), behenic acid (r = −0.47), lignoceric acid (r = −0.53), and phytosterol (r = −0.48). In contrast, Ascomycota was significantly positively correlated with fructose (r = 0.56). Similarly, Basidiomycota was significantly positively correlated with paraoxone (r = 0.53) and lignoceric acid (r = 0.48). Correlation coefficients for the 37 differentially abundant root exudate metabolites and dominant rhizosphere bacterial and fungal phyla are listed in Tables S10 and S11.

## 3. Discussion

Root exudate metabolites vary among plant species, varieties, growth periods, and growth conditions [23]. In the present study, the number of root exudate metabolites with significant differences in pairwise comparisons of the rhizosphere soils of five medicinal plants ranged from 21 (*A. oxyphylla* vs. *A. katsumadai*) to 77 (*A. villosum* vs. *A. katsumadai*), indicating that plant species factors influenced the composition of root exudate metabolites. According to the distinct exudation patterns of 19 *A. thaliana* accessions, root exudate metabolites are affected by the host genotype, with the highest, moderate, and lowest variability observed for glucosinolates, flavonoids, and phenylpropanoids, respectively [24,25]. In the current study, differentially abundant root exudate metabolites among the rhizosphere soils of the five medicinal plants were associated with various KEGG metabolic pathways (metabolic pathways; ABC transporters; glycine, serine and threonine metabolism; and steroid hormone biosynthesis). The root exudate metabolites in the rhizosphere soils of the five medicinal plants and the CK rhizosphere soil were mainly organic acids and derivatives or lipids and lipid-like molecules. Organic acids, which represent a large proportion of exudates, are microbial nutrients, whereas lipids are necessary for mycorrhizal symbiosis; mycorrhizal fungi depend on their hosts for certain lipids [12]. Earlier research indicated that plant exudate metabolites mainly include organic acids and derivatives (wheat, maize, bean, tomato, and beet) or lipids and lipid-like compounds (bean and peanut) [23].

There were no significant differences in the alpha diversity indices of the bacterial and fungal communities among the rhizosphere soils of the five medicinal plants in this study; however, the PCoA results indicated the bacterial and fungal communities of the *A. katsumadai*, *A. officinarum*, *A. oxyphylla*, and *B. cusia* rhizosphere soils were grouped separately from the corresponding communities in the *A. villosum* rhizosphere soil. Furthermore, the *T* test indicated that four dominant phyla in the bacterial communities and one dominant phylum in the fungal communities in terms of relative abundance differed significantly in the pairwise comparisons of the rhizosphere soils of the five medicinal plants. This suggests that different plant species differentially affect the composition and structure of bacterial and fungal communities. A previous study on the effect of plant genotypes on the composition of the rhizosphere microbial community revealed that *A. thaliana* and barley (*Hordeum vulgare*) grown under the same experimental conditions had similar rhizobial communities, with differences in relative abundances and some specific taxonomic groups [26]. The relationship between plant species and rhizosphere microorganisms has been described for Poaceae species [27], rice varieties [28], distant relatives of *A. thaliana* [29], and maize lines [27]. Comparisons with their respective wild relatives have detected distinct rhizosphere microorganisms for domesticated plants, such as maize, barley, *Agave americana*, *Beta vulgaris*, and *Lactuca sativa* [26,30,31,32]. Rovira et al. [33] reported that the nature and quantity of exudate metabolites differed between oat and pea.

Because the ability of rhizosphere microbes to metabolize plant-derived exudate components might determine their success in the microbial community, several studies have investigated the correlation between the diversity of plant exudate metabolites and rhizosphere microbial diversity [12]. Of the metabolites detected in the present study, carvone and zymosterol had opposite effects on Acidobacteria and Actinobacteriota. Similarly, paraoxone and lignoceric acid had the same opposite effects on Ascomycota and Basidiomycota. Different root exudate metabolites may positively or negatively affect rhizosphere microbial communities [34]. A recent study determined that exudate metabolites, such as luteolin and myricetin, had inhibitory effects on some rhizosphere microbes, but had beneficial effects on other microbes [23]. In the current study, two organic acids and derivatives (3-aminoisobutyric acid and 4-acetamidobutyric acid) were positively correlated with Acidobacteria and Actinobacteriota. Some organic acids released by tomato, banana, watermelon, and *A. thaliana* plants may recruit rhizosphere bacteria [20]. Similarly, organic acids secreted by soybean roots significantly promote or inhibit the growth of rhizosphere bacteria, with low and high concentrations typically promoting and inhibiting bacterial growth, respectively [35]. Additionally, in the present study, four and three lipids and lipid-like molecules had positive or negative effects or opposite effects on rhizosphere bacteria and fungi. The results of this study were in accordance with those of previous studies. More specifically, Bi et al. [7] confirmed the significant relationship between lipids and lipid-like compounds and rhizosphere microbial community changes in *Pinus sylvestris* var. *mongolica*. Some phenolic acids (e.g., benzoic acid, caffeic acid, and catechins) may promote or inhibit the breakdown of SOC and affect the microbial community structure [36]. However, these phenolic acids were not detected in the rhizosphere soils of the five medicinal plants in this study, which may be related to the selection of plant and soil types. In addition, as we all know, microalgae are important members of the soil and plant microbiomes, playing a crucial role in maintaining soil and plant health and promoting plant growth [37]. However, unfortunately, there were no microalgae in the results due to the slow update of the database in the annotation of species classification after the sequencing of rhizosphere microorganisms. Even so, we believe that microalgae must exist in the rhizosphere microorganisms in this experiment. We will update the database in the subsequent metagenomic detection of rhizosphere microorganisms, and strive to dig out more types of rhizosphere microorganisms. Furthermore, the bacterial OTUs in the rhizosphere soils of four medicinal plants in this study, with the exception being *A. villosum*, had lower numbers than that of CK. These results showed that root exudates of these four medicinal plants played a more significant role in fine-tuning the composition and function of the rhizosphere bacteria, and inhibited rhizosphere bacterial growth. Root-derived metabolites could recruit or inhibit rhizosphere microorganisms. Previous studies have shown that the toxicity of glucosinolysis products can alter plant-associated growth-promoting microbes and inhibit the excessive proliferation of microbes [38,39].

Only a few compounds, such as coumarins, triterpenoids, and flavonoids, have an established plant root exudate–rhizosphere microbe framework. In one study, the application of *p*-coumaric acid (a well-known component of root exudates) to cucumber seedlings grown in soil led to an increase in the abundances of bacterial and fungal communities. This treatment not only altered the organization and composition of rhizosphere bacterial and fungal communities, but also enhanced the density of a soil-borne pathogen of cucumber, namely *Fusarium oxysporum* f.sp. *cucumerinum* Owen [40]. In addition, the abundance of bacterial groups such as Firmicutes, Betaproteobacteria, and Gammaproteobacteria, as well as fungal groups such as Sordariomycete and Zygomycota, also increased in the cucumber rhizosphere, indicating that these bacterial and fungal groups might be involved in the degradation of *p*-coumaric acid. Another study showed that vanillic acid (a compound secreted by cucumber roots) altered the soil microbial community of cucumber [41]. Similarly, research has shown that activating plant-induced systemic resistance through the induction of jasmonic acid defense pathways significantly alters the rhizosphere microbial community [42]. These findings held significant implications for understanding plant–microbe interactions and how root exudate modulation can influence soil microbial communities. However, most of the plant biosynthetic pathways that have been preliminarily reported to regulate rhizosphere microbial community compositions are still uncharacterized [23]. Our understanding of plant root exudate–microbe interactions will remain incomplete until the molecular basis of the biosynthetic pathways of root-derived specialized metabolites involved in these interactions is elucidated. In future studies, combining transcriptomic, proteomic, and metabonomic tools may facilitate thorough analyses of the molecular mechanisms underlying plant–microbe interactions.

## 4. Materials and Methods

### 4.1. Experimental Sites and Collection of Rhizosphere Samples

Experimental samples were collected on 15 December 2023 from the forest–medicinal planting base, College of Tropical Crops, Yunnan Agricultural University (Pu’er, Yunnan Province, China: latitude 23°06′ N, longitude 101°27′ E, and 1470 m above sea level). The experimental base has a subtropical monsoon climate, with an average annual temperature of 15–20.3 °C, annual frost-free period of more than 315 days, annual rainfall of 1100–2780 mm, and a rainy season from June to October. The region consists mostly of sandy and clay soil. The experimental base is a rubber forest, with most trees more than 10 years old. *A. katsumadai*, *A. villosum*, *A. officinarum*, *A. oxyphylla*, and *B. cusia* plants were grown in the rubber forest for more than 3 years. Fresh *A. katsumadai*, *A. villosum*, *A. officinarum*, *A. oxyphylla*, and *B. cusia* rhizosphere soil samples were used in the experiment, which revealed the effects of root exudate metabolites of different plant species on the rhizosphere microbiota. The soil samples from the pure rubber forest were used as controls in this experiment. Organic and compound fertilizers were not applied to the experimental site. The sampling area of each plant was set up with three parallel plots (15 m × 15 m), with more than 20 m between plots. Three similarly growing plants were randomly selected from similar slopes in each plot. The rhizosphere soils of three plants collected from the same plot were then thoroughly mixed and treated as a biological replicate. The rhizosphere soils of each plant in three experimental plots represented three biological replicates. The same procedure was used to collect non-rhizosphere soil samples (control). Litter as well as herbaceous and shrub humus layers were removed under each plant. Soil less than 5 mm thick that was firmly attached to the surface of the fibrous roots was carefully collected to be used as rhizosphere soil samples. Soil in the pure rubber forest was collected from the same depth as the roots and used as non-rhizosphere soil (control). The collected rhizosphere soil samples were frozen in liquid nitrogen and then stored at −80 °C for the subsequent soil metabolite analysis and DNA extraction.

### 4.2. DNA Extraction and MiSeq Sequencing

Fresh rhizosphere soil DNA was extracted using the E.Z.N.A.^®^ Soil DNA kit (Omega Bio-Tek, Norcross, GA, USA). The 16S rRNA V3–V4 region of the bacterial communities was sequenced using primer pairs 341F (5′-CCTACGGGNGGCWGCAG-3′) and 805R (5′-GactachvgggtattctaATCC-3′) [43]. The ITS2 region of the fungal communities was sequenced using primers ITS1 (5′-GAACCWGCGGARGGATCA-3′) and ITS2 (5′-GCTGCGTTCTTCATCGATGC-3′) [44]. The PCR analysis was repeated three times using a mixture containing 4 μL 5× FastPfu buffer, 2 μL 2.5 mM dNTPs, 0.8 μL each primer (5 μM), 0.4 μL FastPfu polymerase, and 10 ng template DNA. The PCR program was as follows: 95 °C for 3 min; 27 cycles of 95 °C for 30 s, 55 °C for 30 s, 72 °C for 45 s, and 72 °C for 10 min. Amplicons were extracted from a 2% agarose gel, purified using the AxyPrep DNA Gel Extraction kit (Waterman Co., Ltd., London, England), and quantified using QuantiFluor™-ST (Promega (Beijing) Biotech Co., Ltd., Beijing, China). Samples were sequenced on the Illumina NovaSeq platform. The clean data of rhizosphere bacteria and fungi were deposited into the GenBank database (accession number—PRJNA1134174).

### 4.3. Bioinformatic Analyses

Raw sequence data were generated using the QIIME 1.8.0 (Quantitative Insights into Microbial Ecology) pipeline, with different barcodes used for the comparison of all raw reads [45]. Forward and reverse primers were trimmed. A FLASH program was used to combine double-ended reads of sufficient length with overlaps of at least 30 bp to generate a full-length sequence, with an average fragment length of 253 bp [46]. The Btrim program was used to filter low-quality sequences, with a window size greater than 20 for the threshold quality score [47]. UPARSE was used to detect and remove chimeras and assign sequences that were more than 97% similar to the same OTU [48]. The Greengenes database (http://greengenes.lbl.gov/, accessed on 7 March 2024) was used to identify bacteria, whereas the UNITE database (https://unite.ut.ee/, accessed on 7 March 2024) was used to identify fungi. A representative sequence was selected for each OTU. In addition, OTU abundance data were normalized to remove a single OTU from the OTU table. Alpha diversity indices (Observed_OTUs, Chao1, Shannon, and Simpson) were calculated to analyze species diversity and complexity.

### 4.4. Analysis of Root Exudate Metabolites Using a Gas Chromatograph Coupled with a Time-of-Flight Mass Spectrometer

Fresh soil samples (5 g) were placed in a centrifuge tube. Next, 50 mL methanol/hexane/water (3:1:1) solution was added and the centrifuge tube was shaken at 30 °C for 1 h. The sample was then centrifuged at 8000 rpm for 10 min. The supernatant (20 mL) was collected and filtered through a membrane with 0.45 μm pores. The filtered suspension was concentrated to 3–5 mL using a vacuum rotary evaporator and then further concentrated to 1 mL using nitrogen gas. The concentrated suspension was transferred to an Agilent bottle and 100 μL silylated derivatives were added (BSTFA:TCMS = 99:1). The 30 min derivatization process was completed in an incubator set at 60 °C. Methanol (2 mL) was added to dissolve the silanized samples, which were immediately stored at −80 °C prior to the gas chromatography and time-of-flight mass spectrometry (GC-TOF-MS) analysis performed using an Agilent 7890 gas chromatograph coupled with a time-of-flight mass spectrometer. The system included a DB-5MS capillary column. The injection method for each sample involved injecting 1 μL in the splitless mode. The carrier gas was helium, with a purge flow rate of 3 mL min^−1^ for the front inlet and a gas flow rate through the column of 1 mL min^−1^. The initial temperature was held at 50 °C for 1 min, followed by an increase to 310 °C at a rate of 10 °C min^−1^, and then maintained at 310 °C for 8 min. The temperatures of the injection port, transfer line, and ion source were set at 280, 280, and 250 °C, respectively. In the electron impact mode, the energy was set at −70 eV. Mass spectrometry data were acquired in full-scan mode, covering a m/z range of 50–500, at a rate of 12.5 spectra per second, after a solvent delay of 6.4 min. Additionally, the analysis of the raw data, including peak extraction, baseline adjustment, deconvolution, alignment, and integration, was all accomplished through Chroma TOF software (version 4.3x; LECO). During this process, metabolite identification was conducted utilizing the LECO-Fiehn Rtx5 database based on mass spectrometry and retention indices. Finally, the peaks detected in less than half of the QC samples or peaks with RSD >30% in the QC samples were removed.

### 4.5. Statistical Analysis

Data were processed to calculate mean ± standard deviation values using SPSS (version 18.0; IBM Corp., Armonk, NY, USA). The *β*-diversity distance matrix was calculated by QIIME (version 1.8.0) software for sample-level cluster analysis, and then mapped by R language. Principal coordinate analysis (PCoA) was mapped by R language with a weighted unifrac distance matrix using NTSYS pc 2.1 software. The resulting OTU numbers were analyzed by Venn diagrams using the R program. Correlations were determined according to Spearman correlation coefficients. The correlation between the OTU data of rhizosphere microorganisms and root exudate metabolites was determined on the basis of a redundancy analysis using the R package v2.1.

## 5. Conclusions

According to the results of this study, plant root exudate metabolites shape the microbiota in the rhizosphere soils of five medicinal plants. The root exudate metabolites and microbial community structures differed among the rhizosphere soils of the five medicinal plants. The correlations between root exudate metabolites and rhizosphere microbiota indicated that 10 root exudate metabolites belonging to three superclasses (lignans, neolignans, and related compounds; lipids and lipid-like molecules; and organic acids and derivatives) had extremely significant or significant correlations with two dominant rhizosphere bacterial phyla (Acidobacteria and Actinobacteriota) and two dominant rhizosphere fungal phyla (Ascomycota and Basidiomycota). Moreover, two root exudate metabolites (carvone and zymosterol) had opposite effects on rhizosphere bacteria and fungi.

## Figures and Tables

**Table 1:** Dominant phyla of bacterial communities in terms of relative abundance in pairwise comparisons of the rhizosphere soils of *A. katsumadai*, *A. villosum*, *A. officinarum*, *A. oxyphylla*, *B. cusia*, and the pure rubber forest.

Bacterial Phylum	Relative Abundance Comparison between Varieties
Acidobacteria	*A. katsumadai* (33.00 ± 0.69) vs. CK (23.73 ± 0.75)
	*A. officinarum* (17.76 ± 0.24) vs. CK (23.73 ± 0.75)
	*B. cusia* (25.45 ± 0.36) vs. CK (23.73 ± 0.75)
	*A. katsumadai* (33.00 ± 0.69) vs. *A. officinarum* (17.76 ± 0.24)
	*A. katsumadai* (33.00 ± 0.69) vs. *B. cusia* (25.45 ± 0.36)
	*A. katsumadai* (33.00 ± 0.69) vs. *A. villosum* (21.92 ± 1.62)
	*A. katsumadai* (33.00 ± 0.69) vs. *A. oxyphylla* (22.82 ± 1.88)
	*A. officinarum* (17.76 ± 0.24) vs. *B. cusia* (25.45 ± 0.36)
	*A. officinarum* (17.76 ± 0.24) vs. *A. villosum* (21.92 ± 1.62)
	*A. officinarum* (17.76 ± 0.24) vs. *A. oxyphylla* (22.82 ± 1.88)
	*B. cusia* (25.45 ± 0.36) vs. *A. villosum* (21.92 ± 1.62)
	*B. cusia* (25.45 ± 0.36) vs. *A. oxyphylla* (22.82 ± 1.88)
Proteobacteria	*A. katsumadai* (25.80 ± 0.94) vs. CK (19.42 ± 0.67)
	*A. officinarum* (21.99 ± 0.94) vs. CK (19.42 ± 0.67)
	*B. cusia* (22.77 ± 1.84) vs. CK (19.42 ± 0.67)
	*A. katsumadai* (25.80 ± 0.94) vs. *A. officinarum* (21.99 ± 0.94)
	*A. katsumadai* (25.80 ± 0.94) vs. *B. cusia* (22.77 ± 1.84)
	*A. katsumadai* (25.80 ± 0.94) vs. *A. villosum* (21.60 ± 2.74)
	*A. katsumadai* (25.80 ± 0.94) vs. *A. oxyphylla* (19.01 ± 0.80)
	*A. officinarum* (21.99 ± 0.94) vs. *A. oxyphylla* (19.01 ± 0.80)
	*B. cusia* (22.77 ± 1.84) vs. *A. oxyphylla* (19.01 ± 0.80)
Chloroflexi	*A. katsumadai* (13.30 ± 1.04) vs. CK (23.87 ± 1.77)
	*A. officinarum* (28.78 ± 2.09) vs. CK (23.87 ± 1.77)
	*A. villosum* (13.80 ± 1.91) vs. CK (23.87 ± 1.77)
	*A. katsumadai* (13.30 ± 1.04) vs. *A. officinarum* (28.78 ± 2.09)
	*A. katsumadai* (13.30 ± 1.04) vs. *B. cusia* (18.17 ± 1.22)
	*A. katsumadai* (13.30 ± 1.04) vs. *A. oxyphylla* (24.83 ± 0.40)
	*A. officinarum* (28.78 ± 2.09) vs. *B. cusia* (18.17 ± 1.22)
	*A. officinarum* (28.78 ± 2.09) vs. *A. villosum* (13.80 ± 1.91)
	*A. officinarum* (28.78 ± 2.09) vs. *A. oxyphylla* (24.83 ± 0.40)
	*B. cusia* (18.17 ± 1.22) vs. *A. oxyphylla* (24.83 ± 0.40)
	*A. villosum* (13.80 ± 1.91) vs. *A. oxyphylla* (24.83 ± 0.40)
Actinobacteriota	*A. katsumadai* (7.38 ± 0.71) vs. CK (9.16 ± 0.52)
	*A. officinarum* (13.15 ± 1.40) vs. CK (9.16 ± 0.52)
	*B. cusia* (10.13 ± 0.34) vs. CK (9.16 ± 0.52)
	*A. oxyphylla* (11.11 ± 0.93) vs. CK (9.16 ± 0.52)
	*A. katsumadai* (7.38 ± 0.71) vs. *A. officinarum* (13.15 ± 1.40)
	*A. katsumadai* (7.38 ± 0.71) vs. *B. cusia* (10.13 ± 0.34)
	*A. katsumadai* (7.38 ± 0.71) vs. *A. villosum* (9.24 ± 0.53)
	*A. katsumadai* (7.38 ± 0.71) vs. *A. oxyphylla* (11.11 ± 0.93)
	*A. officinarum* (13.15 ± 1.40) vs. *B. cusia* (10.13 ± 0.34)
	*A. officinarum* (13.15 ± 1.40) vs. *A. villosum* (9.24 ± 0.53)
	*A. villosum* (9.24 ± 0.53) vs. *A. oxyphylla* (11.11 ± 0.93)

Dominant phyla of bacterial communities were determined on the basis of a mean relative abundance ≥10%. CK represents the soil sample from the pure rubber forest.

**Table 2:** Dominant phyla of fungal communities in terms of relative abundance in pairwise comparisons of the rhizosphere soils of *A. katsumadai*, *A. villosum*, *A. officinarum*, *A. oxyphylla*, *B. cusia*, and the pure rubber forest.

Fungal Phylum	*p* Value	Relative Abundance Comparison between Rhizosphere Soil Sample Groups
Ascomycota	0.05	*A. katsumadai* (56.43 ± 4.20) vs. CK (64.71 ± 4.10)
	0.05	*B. cusia* (53.97 ± 5.98) vs. CK (64.71 ± 4.10)
	0.05	*A. officinarum* (47.12 ± 4.58) vs. CK (64.71 ± 4.10)
	0.05	*A. officinarum* (47.12 ± 4.58) vs. *A. oxyphylla* (59.75 ± 3.36)
	0.05	*A. officinarum* (47.12 ± 4.58) vs. *A. villosum* (62.12 ± 5.40)
Basidiomycota	0.05	*A. officinarum* (33.86 ± 3.16) vs. CK (19.06 ± 1.01)

Dominant phyla of fungal communities were determined on the basis of a mean relative abundance ≥10%. CK represents the soil sample from the pure rubber forest.

**Table 3:** Alpha diversity indices of the bacterial communities in the rhizosphere soils of *A. katsumadai*, *A. villosum*, *A. officinarum*, *A. oxyphylla, B. cusia*, and the pure rubber forest. CK represents the soil sample from the pure rubber forest.

Samples	Observed_OTUs	Shannon	Simpson	Chao1	Goods_coverage	Pielou_e
*A. katsumadai*	2124.00 ± 145.34 a	9.97 ± 0.08 b	1.00 ± 0.00 a	2124.29 ± 145.50 a	1.00 ± 0.00 a	0.90 ± 0.01 b
*A. villosum*	2733.00 ± 142.50 a	10.53 ± 0.20 a	1.00 ± 0.00 a	2733.29 ± 242.83 a	1.00 ± 0.00 a	0.92 ± 0.01 a
*A. officinarum*	2376.00 ± 99.25 a	10.10 ± 0.30 b	1.00 ± 0.00 a	2378.31 ± 101.24 a	1.00 ± 0.00 a	0.90 ± 0.01 b
*A. oxyphylla*	2580.33 ± 122.02 a	10.24 ± 0.10 ab	1.00 ± 0.00 a	2582.74 ± 122.93 a	1.00 ± 0.00 a	0.90 ± 0.00 b
*B. cusia*	2534.33 ± 133.81 a	10.33 ± 0.04 ab	1.00 ± 0.00 a	2537.24 ± 135.24 a	1.00 ± 0.00 a	0.91 ± 0.01 ab
CK	2539.00 ± 102.89 a	10.18 ± 0.32 ab	1.00 ± 0.00 a	2541.85 ± 106.09 a	1.00 ± 0.00 a	0.90 ± 0.00 b

Different letters in columns indicate significant differences (*p* < 0.05, *n* = 3).

**Table 4:** Alpha diversity indices of the fungal communities in the rhizosphere soils of *A. katsumadai*, *A. villosum*, *A. officinarum*, *A. oxyphylla, B. cusia*, and the pure rubber forest.

Samples	Observed_OTUs	Shannon	Simpson	Chao1	Goods_coverage	Pielou_e
*A. katsumadai*	736.33 ± 15.31 a	7.16 ± 0.32 a	0.98 ± 0.01 a	736.64 ± 15.50 a	1.00 ± 0.00 a	0.75 ± 0.03 a
*A. villosum*	725.00 ± 64.07 a	6.75 ± 0.27 a	0.97 ± 0.01 a	725.27 ± 14.08 a	1.00 ± 0.00 a	0.71 ± 0.05 a
*A. officinarum*	627.33 ± 12.58 a	6.85 ± 0.22 a	0.97 ± 0.01 a	627.39 ± 18.67 a	1.00 ± 0.00 a	0.74 ± 0.03 a
*A. oxyphylla*	706.67 ± 12.76 a	6.94 ± 0.14 a	0.97 ± 0.01 a	707.03 ± 13.00 a	1.00 ± 0.00 a	0.74 ± 0.03 a
*B. cusia*	785.67 ± 81.21 a	7.14 ± 0.24 a	0.97 ± 0.01 a	785.93 ± 18.15 a	1.00 ± 0.00 a	0.75 ± 0.05 a
CK	607.33 ± 39.43 a	6.95 ± 0.16 a	0.97 ± 0.01 a	607.39 ± 39.49 a	1.00 ± 0.00 a	0.76 ± 0.04 a

CK represents the soil sample from the pure rubber forest. Different letters in columns indicate significant differences (*p* < 0.05, *n* = 3).

**Table 5:** Differentially abundant root exudate metabolites in pairwise comparisons of the rhizosphere soils of *A. katsumadai*, *A. villosum*, *A. officinarum*, *A. oxyphylla*, *B. cusia*, and the pure rubber forest.

Groups in Pairwise Comparison	The Root Exudate Metabolites with Relative Contents that Differed by a Factor of 1000 between Rhizosphere Soils	The Number of Up-Regulated Root Exudate Metabolites with Significant Difference	The Number of Down-Regulated Root Exudate Metabolites with Significant Difference
*A. villosum* vs. *A. katsumadai*	0	51	26
*A. oxyphylla* vs. *A. katsumadai*	1	7	14
*B. cusia* vs. *A. katsumadai*	3	34	24
*A. officinarum* vs. *A. katsumadai*	1	46	21
CK vs. *A. katsumadai*	3	43	12
*A. officinarum* vs. *A. villosum*	0	24	28
*A. oxyphylla* vs. *A. villosum*	1	20	49
*B. cusia* vs. *A. villosum*	1	3	14
CK vs. *A. villosum*	4	45	55
*A. oxyphylla* vs. *A. officinarum*	1	13	30
*B. cusia* vs. *A. officinarum*	3	11	12
CK vs. *A. officinarum*	2	30	51
*B. cusia* vs. *A. oxyphylla*	3	27	17
CK vs. *A. oxyphylla*	5	41	18
CK vs. *B. cusia*	5	28	37

CK represents the soil sample from the pure rubber forest.

## Data Availability

Data are contained within the article and Supplementary Materials.

## Supplementary Materials

The following supporting information can be downloaded at https://www.mdpi.com/article/10.3390/ijms25147786/s1.

## References

[1] Trivedi P., Leach J.E., Tringe S.G., Sa T., Singh B.K. (2020). Plant-microbiome interactions: From community assembly to plant health. Nat. Rev. Microbiol..

[2] Philippot L., Raaijmakers J.M., Lemanceau P., van der Putten W.H. (2013). Going back to the roots: The microbial ecology of the rhizosphere. Nat. Rev. Microbiol..

[3] Dilfuza E., Faina K., Shamil V., Laziza G., Zulfiya K., Ben L. (2010). High incidence of plant growth-stimulating bacteria associated with the rhizosphere of wheat grown on salinated soil in Uzbekistan. Environ. Microbiol..

[4] Rodrigo M., Marco K., Irene D.B., Ester D., Menno V.D.V., Schneider J.H.M., Piceno Y.M., Desantis T.Z., Andersen G.L., Bakker P.A.H.M. (2011). Deciphering the rhizosphere microbiome for disease-suppressive bacteria. Science.

[5] Compant S., Samad A., Faist H., Sessitsch A. (2019). A review on the plant microbiome: Ecology, functions, and emerging trends in microbial application. J. Adv. Res..

[6] Huang X.F., Chaparro J.M., Reardon K.F., Zhang R.F., Shen Q.R., Vivanco J.M. (2014). Rhizosphere interactions: Root exudates, microbes, and microbial communities. Botany.

[7] Bi B.Y., Zhang H., Yuan Y., Wu Z.H., Wang Y., Han F.P. (2021). Dynamic changes of soil microbial community in *Pinus sylvestris* var. mongolica plantations in the Mu Us Sandy Land. J. Environ. Manag..

[8] Bai B., Liu W., Qiu X., Zhang J., Zhang J., Bai Y. (2022). The root microbiome: Community assembly and its contributions to plant fitness. J. Integr. Plant Biol..

[9] Xiao X., Chen W.M., Zong L., Yang J., Jiao S., Lin Y.B., Wang E.T., Wei G.H. (2017). Two cultivated legume plants reveal the enrichment process of the microbiome in the rhizocompartments. Mol. Ecol..

[10] Tkacz A., Cheema J., Chandra G., Grant A., Poole P.S. (2015). Stability and succession of the rhizosphere microbiota depends upon plant type and soil composition. ISME J..

[11] Tkacz A., Bestion E., Bo Z., Hortala M., Poole P.S. (2020). Influence of plant fraction, soil, and plant species on microbiota: A multikingdom comparison. mBio.

[12] Sasse J., Martinoia E., Northen T. (2018). Feed your friends: Do plant exudates shape the root microbiome?. Trends Plant Sci..

[13] Bais H.P., Weir T.L., Gilroy S., Vivanco J.M. (2006). The role of root exudates in rhizosphere interactions with plants and other organisms. Annu. Rev. Plant Biol..

[14] Liu Y., Wang H., Peng Z., Li D., Chen W., Jiao S., Wei G. (2021). Regulation of root secondary metabolites by partial root-associated microbiotas under the shaping of licorice ecotypic differentiation in northwest China. J. Integr. Plant Biol..

[15] Liu Y., Li D., Gao H., Li Y., Chen W., Jiao S., Wei G. (2022). Regulation of soil micro-foodwebs to root secondary metabolites in cultivated and wild licorice plants. Sci. Total Environ..

[16] Rodrigo C., Monika G.T., Nicole M., Jana L., Gabriele B., Kornelia S. (2010). Effects of site and plant species on rhizosphere community structure as revealed by molecular analysis of microbial guilds. FEMS Microbiol. Ecol..

[17] Voges M.J.E.E.E., Bai Y., Schulze-Lefert P., Sattely E.S. (2019). Plant-derived coumarins shape the composition of an *Arabidopsis* synthetic root microbiome. Proc. Natl. Acad. Sci. USA.

[18] Huang A.C., Juang T., Liu Y.X., Bai Y.C., Reed J., Qu B.Y., Goossens A., Nutzmann H.-W., Bai Y., Osbourn A. (2019). A specialized metabolic network selectively modulates *Arabidopsis* root microbiota. Science.

[19] Neal A.L., Ahmad S., Gordon-Weeks R., Ton J. (2012). Benzoxazinoids in root exudates of maize attract *Pseudomonas putida* to the rhizosphere. PLoS ONE.

[20] Liu Y.P., Zhang N., Qiu M.H., Feng H.C., Vivanco J.M., Shen Q.R., Zhang R.F. (2014). Enhanced rhizosphere colonization of *beneficial Bacillus amyloliquefaciens* SQR9 by pathogen infection. FEMS Microbiol. Lett..

[21] Zhalnina K., Louie K.B., Hao Z., Mansoori N., da Rocha U.N., Shi S., Cho H., Karaoz U., Loqué D., Bowen B.P. (2018). Dynamic root exudate chemistry and microbial substrate preferences drive patterns in rhizosphere microbial community assembly. Nat. Microbiol..

[22] Zhang C.Y., Lin W.X. (2009). Allelopathic autotoxicity and continuous cropping barriers of medicinal plants. Chin. J. Eco-Agr..

[23] Wang X.C., Zhang J.Y., Lu X.J., Bai Y., Wang G.D. (2024). Two diversities meet in the rhizosphere: Root specialized metabolites and microbiome. J. Genet. Genom..

[24] Mönchgesang S., Strehmel N., Schmidt S., Westphal L., Taruttis F., Muller E., Herklotz S., Neumann S., Scheel D. (2016). Natural variation of root exudates in *Arabidopsis thaliana*-linking metabolomic and genomic data. Sci. Rep..

[25] Mönchgesang S., Strehmel N., Trutschel D., Westphal L., Neumann S., Scheel D. (2016). Plant-to-plant variability in root metabolite profiles of 19 *Arabidopsis thaliana* accessions is substance-class-dependent. Int. J. Mol. Sci..

[26] Bulgarelli D., Garrido-Oter R., Munch P.C., Weiman A., Droge J., Pan Y., McHardy A.C., Schulze-Lefert P. (2015). Structure and function of the bacterial root microbiota in wild and domesticated barley. Cell Host Microbe.

[27] Bouffaud M.-L., Poirier M.-A., Muller D., Moenne-Loccoz Y. (2014). Root microbiome relates to plant host evolution in maize and other Poaceae. Environ. Microbiol..

[28] Edwards J., Johnson C., Santos-Medellin C., Lurie E., Podishetty N.K., Bhatnagar S., Eisen J.A., Sundaresan V. (2015). Structure, variation, and assembly of the root-associated microbiomes of rice. Proc. Natl. Acad. Sci. USA.

[29] Schlaeppi K., Dombrowski N., Oter R.G., Themaat E.V.L.V., Schulze-Lefert P. (2014). Quantitative divergence of the bacterial root microbiota in *Arabidopsis thaliana* relatives. Proc. Natl. Acad. Sci. USA.

[30] Cardinale M., Grube M., Erlacher A., Quehenberger J., Berg G. (2014). Bacterial networks and co-occurrence relationships in the lettuce root microbiota. Environ. Microbiol..

[31] Zachow C., Muller H., Tilcher R., Berg G. (2014). Differences between the rhizosphere microbiome of *Beta vulgaris* ssp maritima—Ancestor of all beet crops—And modern sugar beets. Front. Microbiol..

[32] Coleman-Derr D., Desgarennes D., Fonseca-Garcia C., Gross S., Clingenpeel S., Woyke T., North G., Visel A., Partida-Martinez L.P., Tringe S.G. (2015). Plant compartment and biogeography affect microbiome composition in cultivated and native Agavespecies. New Phytol..

[33] Rovira A.D. (1953). Some quantitative and qualitative aspects of the rhizosphere. Aust. Conf. Soil Sci. Adel..

[34] Lambers H., Mougel C., Jaillard B., Hinsinger P. (2009). Plant-microbe-soil interactions in the rhizosphere: An evolutionary perspective. Plant Soil.

[35] Liljeroth E., Baathe G., Mathiasson I. (1990). Root exudation and rhizoplane bacterial abundance of barley (*Herdeum vulgare* L.) in relation to nitrogen fertilization and root growth. Plant Soil.

[36] van Dam N.M., Bouwmeester H.J. (2016). Metabolomics in the rhizosphere: Tapping into belowground chemical communication. Trends Plant Sci..

[37] Quintas-Nunes F., Brandão P.R., Barreto Crespo M.T., Glick B.R., Nascimento F.X. (2023). Plant growth promotion, phytohormone production and genomics of the rhizosphere-associated microalga, micractinium rhizosphaerae sp. nov. Plants.

[38] Hiruma K., Gerlach N., Sacristán S., Nakano R.T., Hacquard S., Kracher B., Neumann U., Ramirez D., Bucher M., O’Connell R.J. (2016). Root endophyte *Colletotrichum tofieldiae* confers plant fitness benefits that are phosphate status dependent. Cell.

[39] Lahrmann U., Strehmel N., Langen G., Frerigmann H., Leson L., Ding Y., Scheel D., Herklotz S., Hilbert M., Zuccaro A. (2015). Mutualistic root endophytism is not associated with the reduction of saprotrophic traits and requires a noncompromised plant innate immunity. New Phytol..

[40] Zhou X., Wu F. (2012). *p*-Coumaric acid influenced cucumber rhizosphere soil microbial communities and the growth of *Fusarium oxysporum* f.sp. *cucumerinum* Owen. PLoS ONE.

[41] Zhou X., Wu F. (2013). Artificially applied vanillic acid changed soil microbial communities in the rhizosphere of cucumber (*Cucumis sativus* L.). Can. J. Soil Sci..

[42] Carvalhais L.C., Dennis P.G., Badri D.V., Tyson G.W., Vivanco J.M., Schenk P.M. (2013). Activation of the jasmonic acid plant defence pathway alters the composition of rhizosphere bacterial communities. PLoS ONE.

[43] Evans S.E., Wallenstein M.D., Burke I.C. (2014). Is bacterial moisture niche a good predictor of shifts in community composition under long-term drought?. Ecology.

[44] Gardes M., Bruns D. (1993). ITS primers with enhanced specificity for basidiomycetes-application to the identification of mycorrhizae and rusts. Mol. Ecol..

[45] Caporaso J.G., Kuczynski J., Stombaugh J., Bittinger K., Bushman F.D., Costello E.K., Fierer N., Pena A.G., Goodrich J.K., Gordon J.I. (2010). QIIME allows analysis of highthroughput community sequencing data. Nat. Methods.

[46] Reyon D., Tsai S.Q., Khayter C., Foden J.A., Sander J.D., Joung J.K. (2012). FLASH assembly of TALENs for high-throughput genome editing. Nat. Biotechnol..

[47] Yong K. (2011). Btrim: A fast, lightweight adapter and quality trimming program for next generation sequencing technologies. Genomics.

[48] Edgar R.C. (2013). UPARSE: Highly accurate OTU sequences from microbial amplicon reads. Nat. Methods.

