# Supplementary material for: Medicinal Plant Root Exudate Metabolites Shape the Rhizosphere Microbiota
Source: Int J Mol Sci. 2024 Jul 16;25(14):7786. doi: 10.3390/ijms25147786 (PMC11277521; doi:10.3390/ijms25147786)
Supplement: Supplementary file 1 [file ijms-25-07786-s001.zip › Figure S1.pdf]

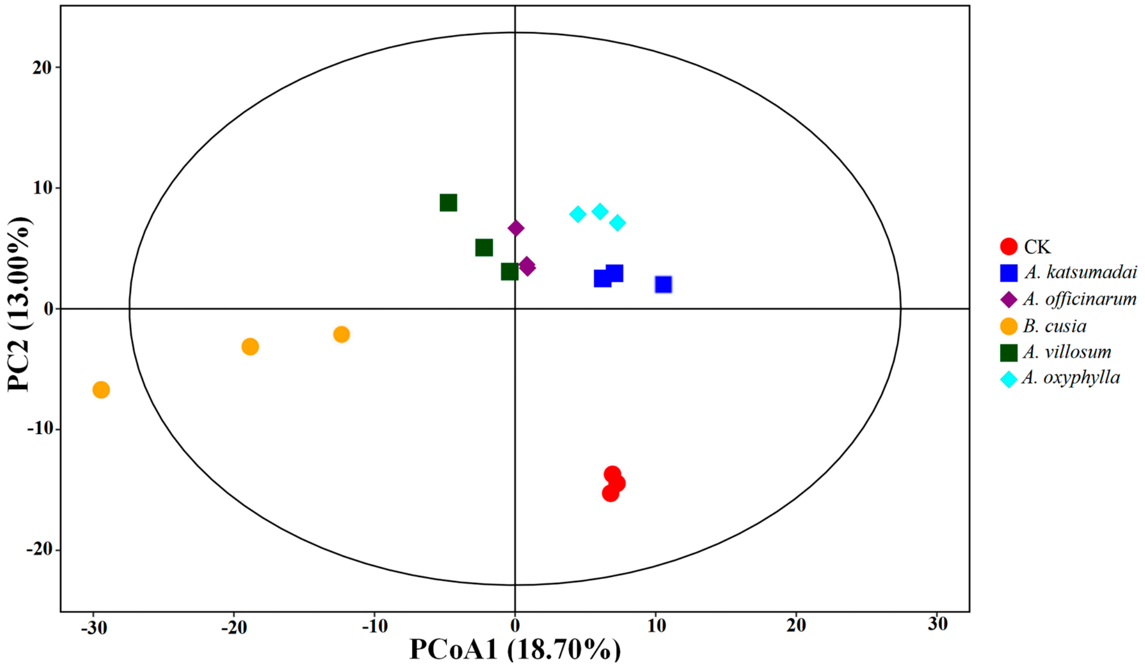

Figure S1. PCA scores plot for root exudate metabolites among the rhizosphere soils of *A. katsumadai*, *A. villosum*, *A. officinarum*, *A. oxyphylla*, *B. cusia*, and CK.
