# Supplementary material for: Medicinal Plant Root Exudate Metabolites Shape the Rhizosphere Microbiota
Source: Int J Mol Sci. 2024 Jul 16;25(14):7786. doi: 10.3390/ijms25147786 (PMC11277521; doi:10.3390/ijms25147786)
Supplement: Supplementary file 1 [file ijms-25-07786-s001.zip › Figure S2.pdf]

Figure S2. Overview of the analysis of differentially abundant root exudate metabolites in pairwise comparisons of the rhizosphere soils of *A. katsumadai*, *A. villosum*, *A. officinarum*, *A. oxyphylla*, *B. cusia*, and the pure rubber forest. The horizontal coordinate presents the DA score, whereas the vertical coordinate presents the KEGG metabolic pathway. The DA score reflects the overall change in all metabolites associated with a metabolic pathway. A score of 1 indicates that all annotated differentially abundant metabolites in the pathway are up-regulated, whereas a score of −1 indicates that all annotated differentially abundant metabolites in the pathway are down-regulated. The length of the line segment indicates the absolute DA score. The size of the dot indicates the number of differentially abundant metabolites associated with the pathway. The dot distribution on the right side of the central axis and a long line segment indicate that the metabolites associated with the pathway tend to be up-regulated. The dot distribution on the left side of the central axis and a long line segment indicate the metabolites associated with the pathway tend to be down-regulated. * significant difference ($p < 0.05$); ** extremely significant difference ($p < 0.01$); *** extremely significant difference ($p < 0.001$)

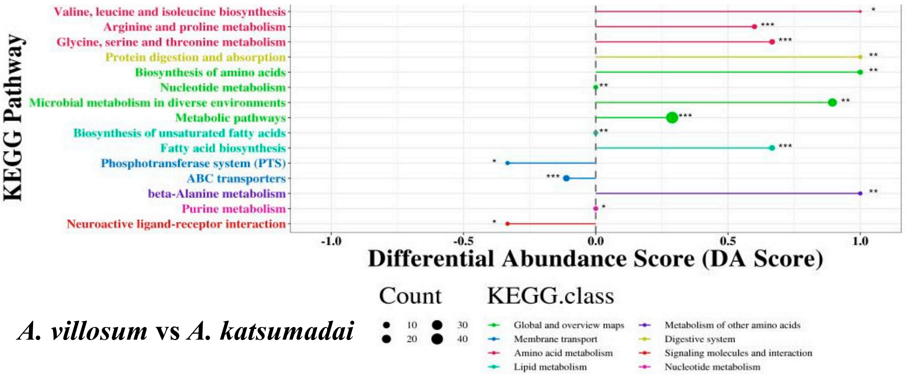

A. villosum* vs *A. katsumadai

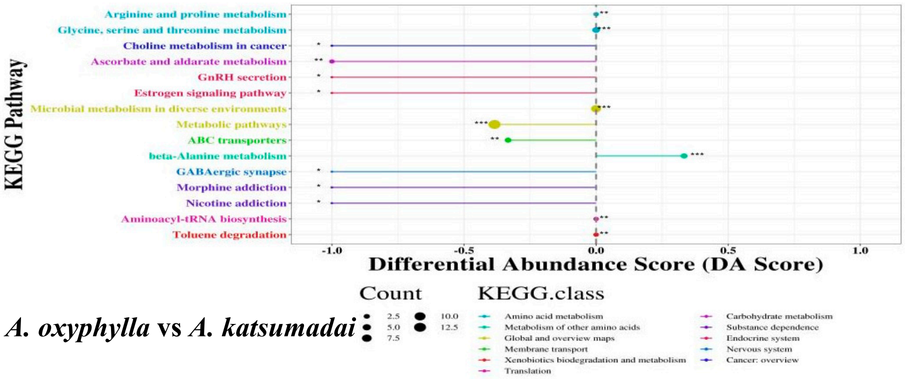

A. oxyphylla* vs *A. katsumadai

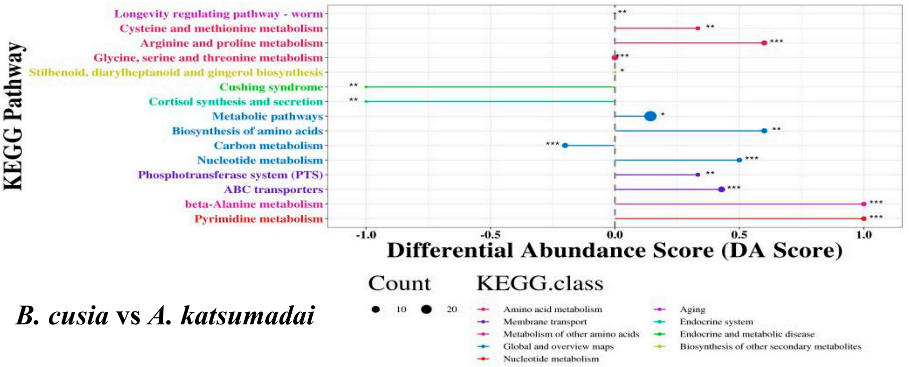

B. cusia* vs *A. katsumadai

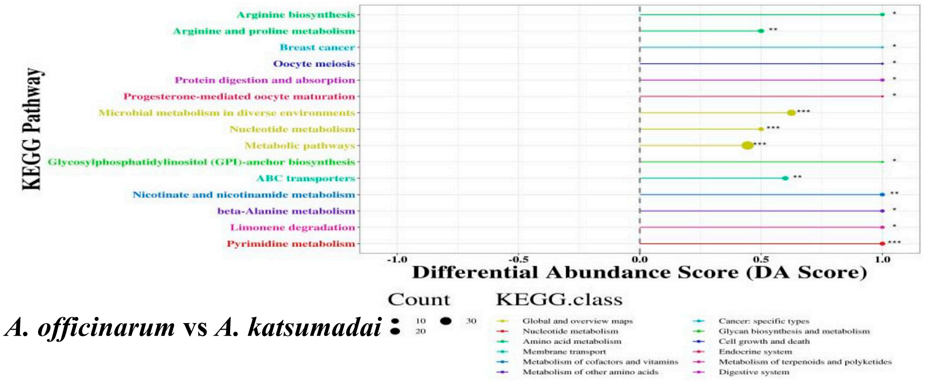

A. officinarum* vs *A. katsumadai

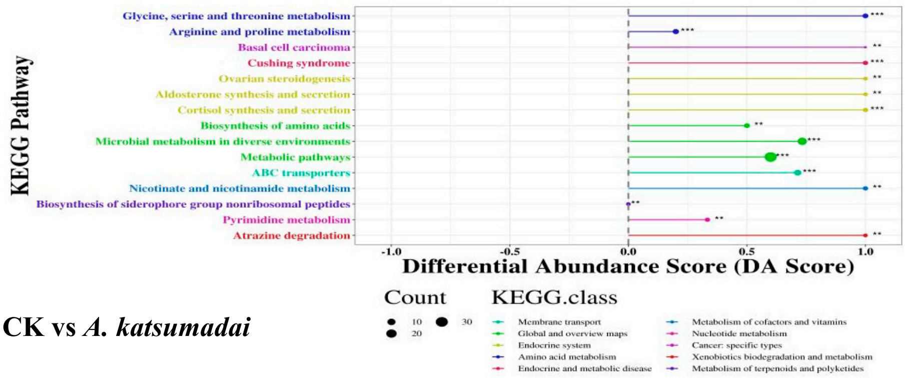

CK vs *A. katsumadai*

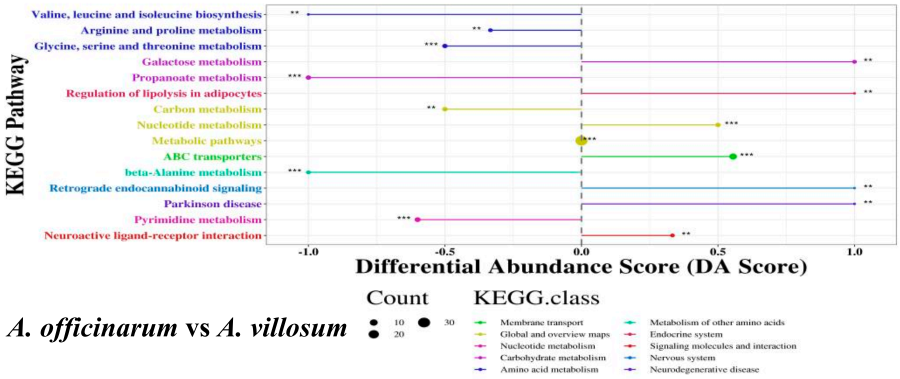

A. officinarum* vs *A. villosum

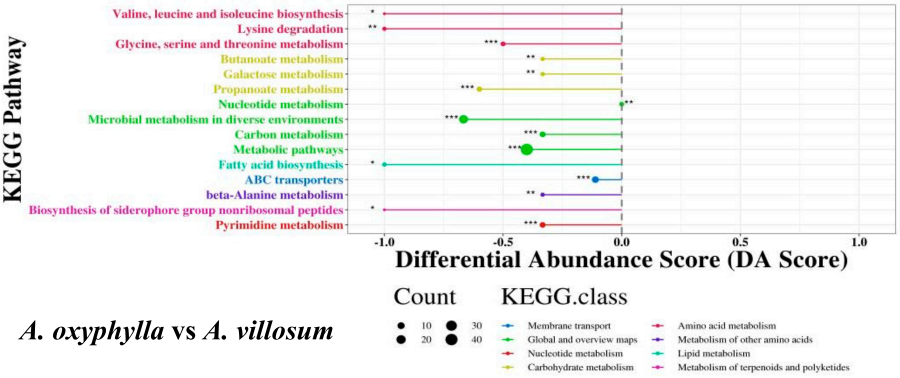

A. oxyphylla* vs *A. villosum

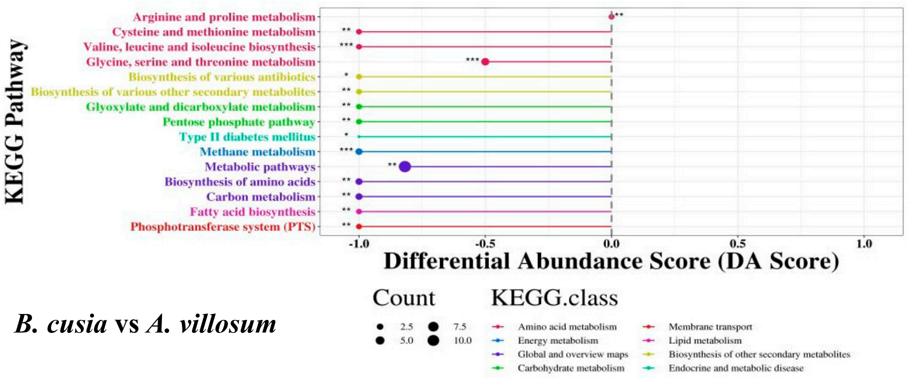

B. cusia* vs *A. villosum

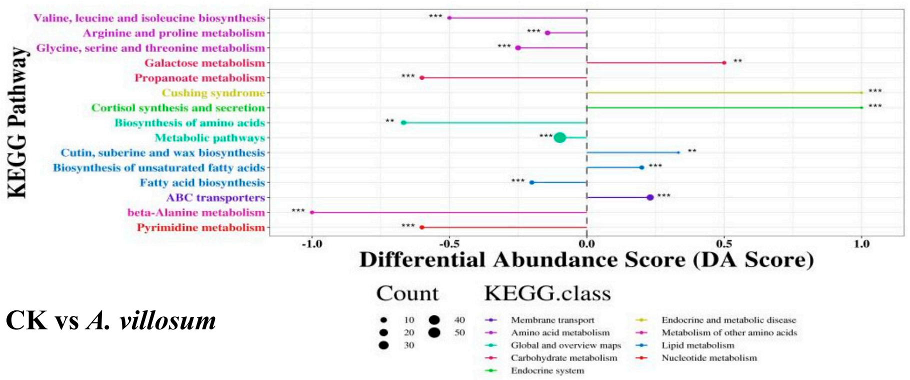

CK vs *A. villosum*

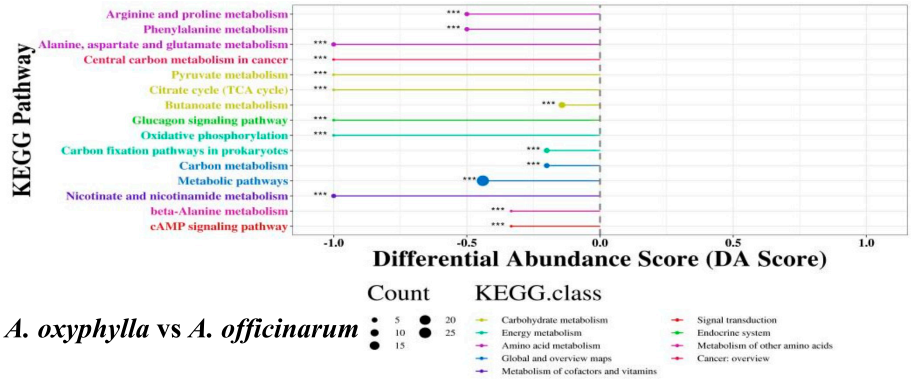

A. oxyphylla* vs *A. officinarum

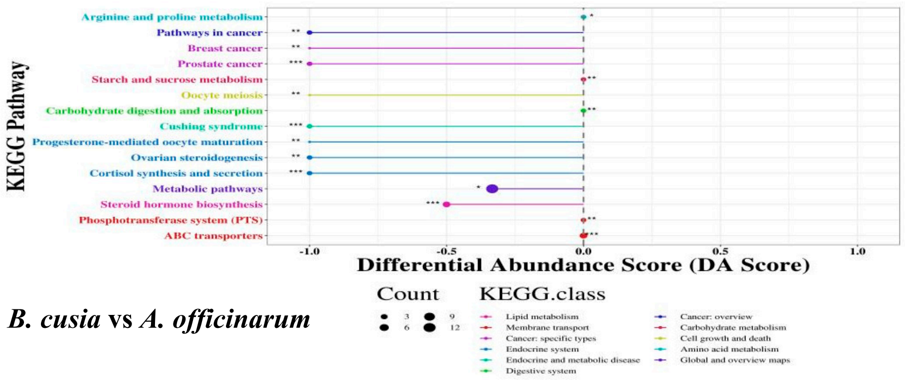

B. cusia* vs *A. officinarum

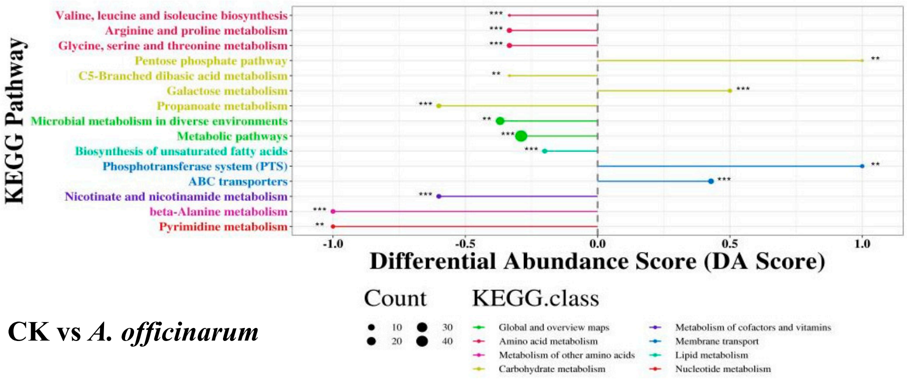

CK vs *A. officinarum*

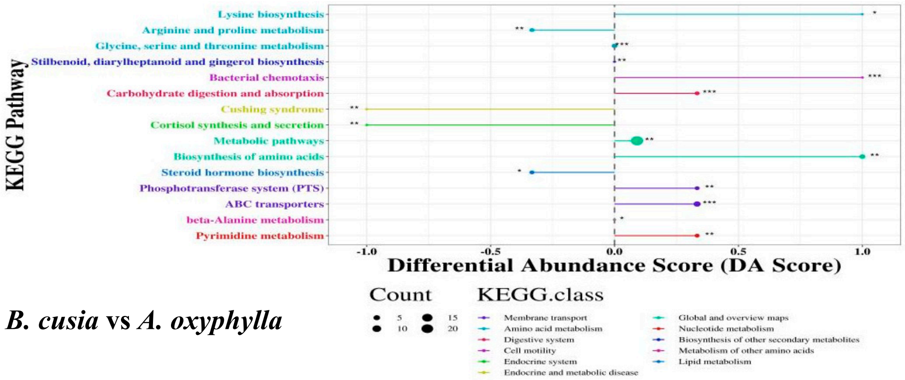

B. cusia* vs *A. oxyphylla

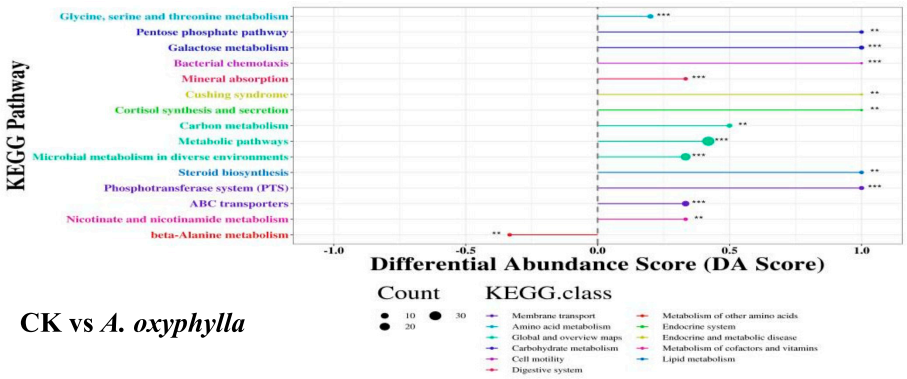

CK vs *A. oxyphylla*

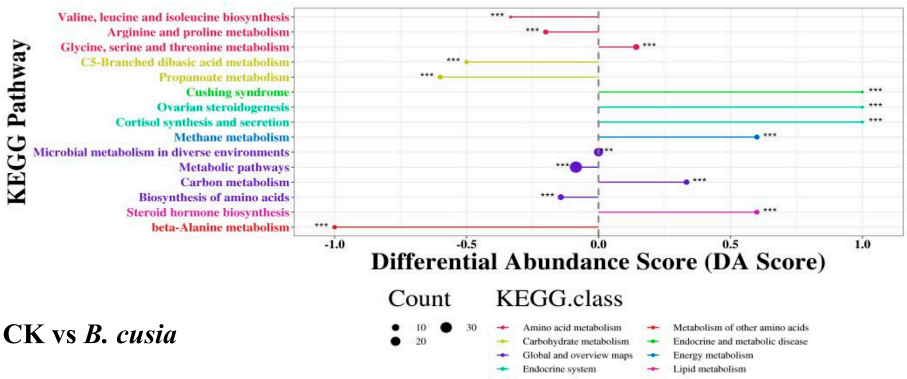

CK vs *B. cusia*
